# An expert-led and artificial intelligence system-assisted tutoring course to improve the confidence of Chinese medical interns in suturing and ligature skills: a prospective pilot study

**DOI:** 10.3352/jeehp.2019.16.7

**Published:** 2019-04-10

**Authors:** Ying-Ying Yang, Boaz Shulruf

**Affiliations:** 1 Division of Clinical Skills Training and High-fidelity Medical Simulation for Holistic Care and Inter-Professional Collaboration, Department of Medical Education, Taipei Veterans General Hospital, Taipei, Taiwan; 2 Department of Medicine, National Yang-Ming University, Taipei, Taiwan; 3 Division of General Medicine, Department of Medicine, Taipei Veterans General Hospital, Taipei, Taiwan; 4 Office of Medical Education, University of New South Wales Australia, Sydney, Australia; Hallym University, Korea

**Keywords:** Artificial intelligence, Suturing and ligature skills, Tutoring course, Taiwan

## Abstract

**Purpose:**

Lack of confidence in suturing/ligature skills due to insufficient practice and assessments is common among novice Chinese medical interns. This study aimed to improve the skill acquisition of medical interns through a new intervention program.

**Methods:**

In addition to regular clinical training, expert-led or expert-led plus artificial intelligence (AI) system tutoring courses were implemented during the first 2 weeks of the surgical block. Interns could voluntarily join the regular (no additional tutoring), expert-led tutoring, or expert-led+AI tutoring groups freely. In the regular group, interns (n=25) did not receive additional tutoring. The expert-led group received 3-hour expert-led tutoring and in-training formative assessments after 2 practice sessions. After a similar expert-led course, the expert-led+AI group (n=23) practiced and assessed their skills on an AI system. Through a comparison with the internal standard, the system automatically recorded and evaluated every intern’s suturing/ligature skills. In the expert-led+AI group, performance and confidence were compared between interns who participated in 1, 2, or 3 AI practice sessions.

**Results:**

The end-of-surgical block objective structured clinical examination (OSCE) performance and self-assessed confidence in suturing/ligature skills were highest in the expert-led+AI group. In comparison with the expert-led group, the expert-led+AI group showed similar performance in the in-training assessment and greater improvement in the end-of-surgical block OSCE. In the expert-led+AI group, the best performance and highest post-OSCE confidence were noted in those who engaged in 3 AI practice sessions.

**Conclusion:**

This pilot study demonstrated the potential value of incorporating an additional expert-led+AI system–assisted tutoring course into the regular surgical curriculum.

## Introduction

Suturing/ligature skills are among the most essential foundations on which all surgical skills are built. Development an unconsciously mastered suture skills need repeated practice and years of experience. Nonetheless, incoming interns have a much shorter time to practice suturing and therefore find it difficult to achieve sufficient levels of competence and mastery [1,2]. It is well established that evaluation decreases the learning time and improves the acquisition of suturing skills by medical students [3]. Dehabadi et al. [4] and Botden et al. [5] reported that a computer-enhanced training and feedback system could shorten the maturation time of technical skills. Accordingly, it is important to consider how to teach, evaluate, and provide feedback effectively to ensure interns’ competency in suturing/ligature skills [6,7]. Both peer and expert tutoring promote self-directed learning and motivate interns to perform suturing by consciously recalling suturing steps [8-10]. Simulation has become an increasingly important aspect of surgical education and training [11]. Surgical simulation tutoring offers trainees the opportunity to improve their skills by practicing outside the operating room and compensates for obstacles to developing these skills [12]. Additionally, suturing and performing ligatures require high-level skills in order not to damage skin or issues. Therefore, a training model that objectively evaluates the suturing/ligature skills of novice interns is crucial for enhancing their proficiency.

In current study, our new intervention program provided additional suturing/ligature tutoring for novice interns who had not previously received the corresponding training. Specifically, the medical interns were offered tutoring using 3 different teaching techniques: regular clinical training (the regular group), regular training plus an additional expert-led tutoring course (the expert-led group), and regular training plus additional expert-led+artificial intelligence (AI) tutoring courses (the expert-led+AI group). The primary goal of this study was to compare the relative suturing/ligature skills of the 3 different groups of novice medical interns.

## Methods

### Subjects and methods

Taipei Veteran General Hospital (Taipei VGH) is a medical center that is certified as the formal teaching hospital of National Yang-Ming Medical University. Currently, the curriculum at National Yang-Ming Medical University and Taipei VGH consists of 6 years of teaching, including 4 years of university teaching (first and second years for the basic common course and third and fourth years for the preclinical course) and 2 years of clinical training (internship, fifth and sixth years), which are followed by 2 years of postgraduate residency and several years of sub-specialty residency training.

In detail, the third and fourth (pre-clinical) years are different from the first and second years (basic common course) because they emphasize pre-clinical content, including skills associated with patient encounters, such as history taking, physical examinations, communication, self-directed learning, and problem solving. In the clinical years (internship), students participate in internal medicine, surgery, obstetrics and gynecology, and pediatrics blocks in fifth year and then primary care in their sixth year during clinical rotations.

### Regular method of training suturing/ligature skills before this new intervention program

In the regular method of training suturing/ligature skills, before this new intervention program, medical interns learned their suture and ligature skills by chance from mentors during clinical rotations. Then, interns received feedback from experts after participation in their end-of surgical block formative objective structured clinical examination (OSCE). In the sixth year, medical interns need to develop their suturing/ligature skills continuously in order to pass their final summative OSCE before graduation. In this study, a new intervention program was implemented in the surgical block in the fifth year of clinical training by providing additional expert-led or expert-led plus AI tutoring for suture and ligature skills. The first (regular) group in this study received regular clinical training.

### Ethics statement

This study received ethical approval from the Taipei VGH (approval no., 2017-06-010AC). Informed consent was given before participation in the study.

### Groupings

**Fig. 1.**
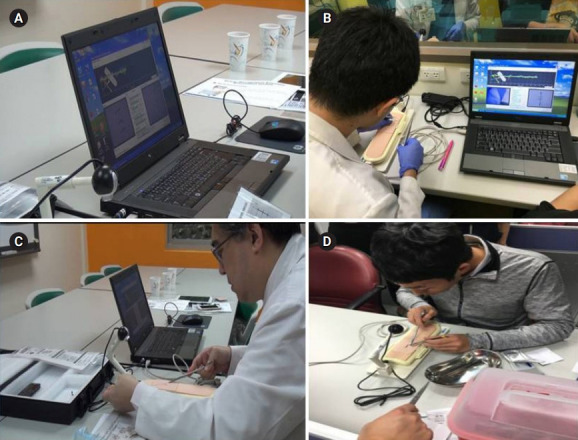
(A–D) Photo of the AI assessment/training system for suturing/ligature skills and images of medical interns practicing.

**Fig. 2.**
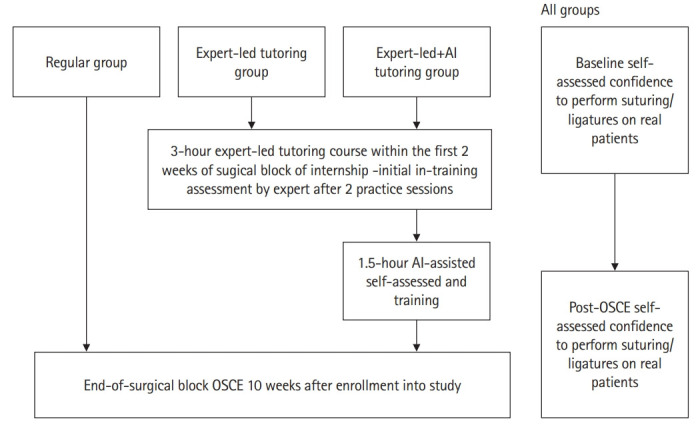
Schematic diagram of the schedule for trainings and assessments of the regular, expert-led tutoring, and expert-led+AI tutoring groups. AI, artificial intelligence; OSCE, objective structured clinical examination.

Between November 2017 and December 2018, novice medical interns who had not received basic suturing/ligature skills training were invited to join this new intervention program. All interns from the classes of 2017 had the opportunity to voluntarily join either the expert-led tutoring (second) or expert-led plus AI (third) tutoring groups during their 3-month surgical block. The first group was interns who did not join either the second or third group; these individuals formed the regular group. Notably, medical interns in the 3 groups received similar regular clinical training in the surgical block. The first (regular, n=25) group received regular clinical training, the second (expert-led, n=24) group received regular training plus additional expert-led tutoring, and the third (expert-led+AI, n=23) group received regular training plus an expert-led+AI course. Notably, the duration of additional tutoring was similar between the expert-led and expert-led+AI tutoring groups (Figs. 1, 2).

### Additional common course for the expert-led and expert-led+AI tutoring groups within first 2 weeks of the surgical block

The common tutoring for both groups began with an orientation on how to suture/ligature on artificial skin by experienced experts (faculty members of the surgical department) according to the objective structural assessment of technical skills (OSATS) criteria for the surgical OSCE. The suturing training kits contained a commercial needle holder, forceps, scissors, needle, silk, and a suturing pad. At the beginning of the tutoring course, the criteria for good suturing, including safety (holding the needle correctly, including posture, grasping the suturing needle with the needle holder approximately one-third of the distance from the eye of the needle, use of the needle holder to pull the needle up from the wound ridge/turning the needle without using the hand to avoid needle stick, and performing a ligature with square knot), quality (avoiding defects or errors in suturing or ligatures) and efficiency (completing 3 vertical mattress sutures within 10 minutes), were given as checklist to each intern. After 2 rounds of practice under expert supervision, following the criteria of the OSATS checklist, an in-training assessment was performed to evaluate the initial effects of expert-led tutoring. The OSATS checklist emphasized the technical performance (TP) score, including a 3-point (1=poor, 2=fair, 3=good) scale for the aforementioned criteria of safety, quality and efficiency as well as the global rating (GR) score (1–5 scale: 1=very poor, 2=poor, 3=average, 4=good, and 5=excellent). For each intern, the maximal TP and GR scores, which would translate into a 100% score, were 9 and 5, respectively. Finally, the expert concluded the teaching session by providing each intern one-on-one feedback according to their performance score at the OSCE station [13]. It was emphasized that participation in the tutoring course was voluntary, and that it would not affect their grade standing.

### Expert-led+AI group-specific AI tutoring

In the expert+AI tutoring group, in addition to receiving a similar course to that of the expert-led group, every intern practiced their skills once on artificial skin that was connected to an AI recording and analysis system for suture/ligature training (Waseda-Kyoto-Kagaka suture no. 2 refined II [WKS-2RII]; Kyoto-Kagaku, Kyoto, Japan). Before practicing, each intern was required to watch the demonstration video. Within 1.5 hours, this AI training system automatically provided qualitative information about each intern’s TP score as a summation of 6 aspects including the skills of time (T, from the start through completion of the task), force in tissue (FoT), judging tension (JuT), equidistance (EqD), distance between sutures (DbS), and wound dehiscence (WoD). Notably, these 6 aspects covered the 3 domains of good suturing/ligature skills: safety (JuT, EqD, WoD), quality (FoT, DbS), and efficiency (T). Clearly, this system emphasized the safety and quality of suturing/ligature skills.

In this study, the WKS-2RII system served as a self-assessment and training tool. The performance scores of each intern were calculated by comparison (percentage) with the performance of novice learners that had been set as the internal standard in the system. A total TP score greater than 70 was considered to be a passing score on the self-assessment, and a total score less than 70 was considered to be a failing score. Thus, every intern received their TP score and pass/fail data from the WKS-2RII system. Then, they used the data as the basis for a discussion with their clinical instructors.

In addition to providing instructions on performing the task, the WKS-2RII system provided learners with images and reference parameters for experienced experts and novice beginners. Through independent practice, medical interns were able to compare their work with these internal standards. The WKS-2RII system served both as a tool for modeling steps of the task and as a reference for the desired outcome, facilitating self-directed learning.

In addition to the minimal requirement of using the AI system for 1.5 hours, the interns in the expert+AI group were encouraged to utilize the AI (WKS-2RII) system as frequently as possible in order to enhance their suturing/ligature skills. The performance of those who had engaged in 1, 2, or 3 practice sessions with the AI system on the end-of-surgical block 1 station OSCE was compared.

### End-of-surgical block OSCE for the 3 groups

Ten weeks after enrollment in the study, the performance of the interns from the 3 groups was assessed through an end-of-surgical block formative OSCE using the same checklist as the in-training assessment. The TP and GR scores were assessed by experienced examiners.

### Inter-rater reliability

At the beginning of the in-training assessment and end-of-surgical block formative OSCE, raters discussed and agreed upon suturing/ligature criteria. A pilot study was conducted using 10 volunteer interns who each performed 3 single sutures.

### Self-assessed confidence on suturing and ligature skills

Using a 1-item questionnaire, self-assessed confidence on performing suturing/ligature skills on real patients was evaluated immediately after enrollment and at the end-of-surgical block OSCE with a 5-point Likert scale (1–5 scale; 1=very unconfident, 3=neutral, and 5=very confident).

### Study design

Among the regular, expert-led, and expert-led+AI groups, this prospective comparative study evaluated the self-assessed confidence and performance of medical interns in performing suturing/ligatures at the end-of-surgical block OSCE.

### Statistics

Data were expressed as mean±standard deviation. A significance level of P=0.05 was chosen. One-way analysis of variance was performed to compare the group characteristics and the assessment data of the regular, expert-led tutoring, and expert-led+AI tutoring groups.

## Results

The basic characteristics of the interns in the 3 groups are shown in Table 1. The mean age, gender distribution, proportion of participants with previous experience of observing suturing/ligature skills on real patients, and baseline self-assessed confidence in performing suturing/ligatures on real patients were not significantly different among the 3 groups. Notably, the degree of improvement in confidence in these skills at the end-of-surgical block OSCE was higher in the expert-led and expert-led+AI tutoring groups than in the regular group. The greatest improvement was noted in the expert-led+AI tutoring group (Table 1). The raw data are available in Supplement 1.

### Additional tutoring significantly enhanced the performance of interns in suturing/ligature skills

Notably, the percentage of interns with a baseline high level of interest in surgery was not significantly different across the 3 groups (Table 2). Meanwhile, the initial TP and GR performance scores after the expert-led tutoring course were similar between the expert-led and expert-led+AI tutoring groups. In general, the performance of the expert-led and expert-led+AI tutoring groups on the end-of-surgical block OSCE was better than that of the regular group. A significantly greater percentage of interns in the expert-led+AI tutoring group showed improvements in their performance than in the expert-led tutoring group.

### Increased frequency of practice on the AI system improved the confidence of interns in their suturing/ligature skills

As shown in Table 3, the average TP scores and pass rates at the last practice session of the expert-led+AI group interns was higher among those who had practiced 3 times on the AI self-assessed training system than among those who had practiced 2 times. Increased frequency of practicing suturing/ligature skills on the AI self-assessed training system significantly improved the confidence of interns in the expert-led+AI group.

## Discussion

Suturing and performing ligatures are the most challenging basic skills for general practitioners in medical training. Repetitive deliberate practice is important for learning surgical and ligature skills [14]. Detailed training and assessment of suturing/ligature skills in the safety, quality, and efficiency domains will improve the confidence of novice learners. Rufai et al. [1] reported that 23% of medical interns had paid for additional surgical skills tutoring to improve their confidence in suturing and performing ligatures. Accordingly, an additional tutoring course was provided to medical interns at our hospital to increase their confidence in suturing/ligature skills before real-world practice.

Typically, the basic instruction provided to medical interns focuses on performing ‘good’ sutures and ties on an OSCE. Without a suitable assessment system, it is difficult to instruct novice learners about which steps they do not do or about deviations from the standard procedure. In fact, some specific elements, including force in tissue, judging tension, equidistance between sutures, and wound dehiscence of sutures and ties, are difficult to check by direct observation of the task. In other words, those aforementioned details are not easily assessed with the OSATS criteria on an OSCE by the simple observation. In contrast, the AI system in our study separated the skills of suturing and performing ligatures into specific steps and highlighted all important steps. Therefore, medical interns were able to monitor their progress in developing these skills by using this AI system. Thus, the expert-led+AI tutoring course is an acceptable strategy for training medical interns in suturing and ligature skills. By engaging learners in the training, teachers can involve themselves in the educational program in different ways (for example, by organizing more comprehensive courses).

According to the classic learning theory, detail information helps navíe students to learn complex task after efficient processing [15]. Meanwhile, learning theory indicates that a trainee must progress through cognitive, integrative, and autonomous stages [16]. Proficiency-based training emphasizes self-directed practice to achieve a predetermined level of proficiency. Accurate self-assessment is crucial both to promote self-efficiency and to prevent overconfidence or distress. Accordingly, the WKS-2RII system can cultivate learners to the point of autonomously performing suturing and ligatures proficiently.

A limitation of this study is that it was a small-scale pilot study only implemented at a single site. The performance of the medical interns in the regular group on the end-of-surgical block OSCE reflected the actual real-world learning of suturing/ligature skills. Additional tutoring, in either the expert-led or expert-led+AI tutoring group, will benefit medical interns in their surgical block. Therefore, the better performance of the expert-led and expert-led+AI tutoring groups may be attributable to the additional training these groups had in comparison with the regular group. Furthermore, in this study, interns voluntarily joined either the additional expert-led or the expert-led+AI courses. It is expected that these voluntary medical interns had higher motivation for learning than others. Therefore, it is possible that the better performance of the intervention groups might have at least partially stemmed from their higher intrinsic motivation for learning. Accordingly, the better performance of the expert-led and expert-led+AI tutoring groups reflects the combined effects of additional training and intrinsic motivation of the medical interns who joined those groups voluntarily.

Interestingly, the generally low level of baseline confidence in suturing and ligature skills across the regular, expert-led, and expert-led+AI groups indicates that tutoring is necessary for interns. Additionally, the relatively poor performance of the regular group on the end-of-block surgical OSCE indicates that additional tutoring, either through an expert-led course or an expert-led+AI course, should be included in the regular curriculum.

In conclusion, expert-led and AI-assisted tutoring is a feasible teaching method for novice medical interns, and yielded progressive improvements in their performance scores on suturing/ligature skills. Increasing the frequency of practice with the AI system improved medical interns’ confidence and performance in suturing/ligature skills. This study suggests that suturing/ligature tutoring should be implemented in parallel with real patient practice to increase medical interns’ confidence in suturing/ligature skills.

## Figures and Tables

**Table 1.** Baseline characteristics of all medical interns

Characteristic	Regular group (n=25)	Expert-led tutoring group (n=24)	Expert-led+AI group (n=23)
Mean age (yr)	27±4	28±2	29±3
Gender distribution (male/female)	14/11	12/12	12/11
Prior experience of observing suturing/ligature skills on real patients (%)	10/25 (40)	11/24 (46)	11/23 (48)
Baseline self-assessed confidence to perform suturing/ligatures on real patients (points out of 5)	2.4±0.5	2.6±0.2	2.5±0.1
Post-objective structured clinical examination self-assessed confidence to perform suturing/ligatures on real patients (points out of 5)	2.7±0.1	3.4±0.3	4.0±0.5
Percentage showing improvement in self-assessed confidence from baseline (%)	15	31	60

Values are presented as mean±standard deviation or number, unless otherwise stated. AI, artificial intelligence.

**Table 2.** Comparison of the performance of medical interns among groups

Variable	Regular group (n=25)	Expert-led tutoring group (n=24)	Expert-led+AI tutoring group (n=23)
Percentage of those who had a high level of interest (>3 points out of 5) in surgery at baseline (%)	8/25 (32)	8/24 (33)	9/23 (39)
Performance of initial in-training assessment by expert			
Technical performance	-	69±8	70±5
Global rating	-	3.4±0.5	3.6±0.2
Performance on common end-of-surgical block objective structured clinical examination			
Technical performance	71.5±3	79.1±4 (15% increase from baseline)	90.2±2^a),b)^ (30% increase from baseline)
Global rating	3.7±0.3	4.0±0.6 (18% increase from baseline)	4.5±0.9^a),b)^ (25% increase from baseline)

Values are presented as number (%) or mean±standard deviation, unless otherwise stated. AI, artificial intelligence.

a) P<0.05 vs. expert-led tutoring group’s interns.

b) P<0.05 vs. regular group’s medical interns.

**Table 3.** Average performance of interns in the expert-led+AI group on their last practice session according to frequency of practice with the AI system

Variable	Technical performance score	Pass rate (%)	Follow-up self-assessed confidence to perform suturing and ligatures on real patients
1 Practice session (n=23)	69.4±12	25.00±3.7	3.8±0.3
2 Practice sessions (n=13)	80.4±9^a)^	55.00±3.2^a)^	4.0±0.12
Absolute increase from baseline (%)	11.08	30	0.2
3 Practice sessions (n=6)	87.1±8^a)^	81.00±3.8^a),b)^	4.7±1.2^a)^
Absolute increase from baseline (%)	17.7^a)^	56^a)^	0.9

Values are presented as mean±standard deviation or number, unless otherwise stated. AI, artificial intelligence.

a) P<0.05 vs. the 1-practice group.

b) P<0.05 vs. the 2-practice group.

## Supplementary materials

Supplement 1. Data files are available from https://doi.org/10.7910/DVN/WMFPJ8.

Supplement 2. Audio recording of the abstract.
